# An Outline of Renal Artery Stenosis Pathophysiology—A Narrative Review

**DOI:** 10.3390/life11030208

**Published:** 2021-03-07

**Authors:** Lukasz Dobrek

**Affiliations:** Department of Clinical Pharmacology, Wroclaw Medical University, Wroclaw, Poland; lukasz.dobrek@umed.wroc.pl; Tel.: +48-71-7840601

**Keywords:** renal artery stenosis, ischemic nephropathy, renovascular hypertension, pathophysiology

## Abstract

Renal artery stenosis (RAS) is conditioned mainly by two disturbances: fibromuscular dysplasia or atherosclerosis of the renal artery. RAS is an example of renovascular disease, with complex pathophysiology and consequences. There are multiple pathophysiological mechanisms triggered in response to significant renal artery stenosis, including disturbances within endothelin, kinin–kallikrein and sympathetic nervous systems, with angiotensin II and the renin–angiotensin-aldosterone system (RAAS) playing a central and key role in the pathogenesis of RAS. The increased oxidative stress and the release of pro-inflammatory mediators contributing to pathological tissue remodelling and renal fibrosis are also important pathogenetic elements of RAS. This review briefly summarises these pathophysiological issues, focusing on renovascular hypertension and ischemic nephropathy as major clinical manifestations of RAS. The activation of RAAS and its haemodynamic consequences is the primary and key element in the pathophysiological cascade triggered in response to renal artery stenosis. However, the pathomechanism of RAS is more complex and also includes other disturbances that ultimately contribute to the development of the diseases mentioned above. To sum up, RAS is characterised by different clinical pictures, including asymptomatic disorders diagnosed in kidney imaging, renovascular hypertension, usually characterised by severe course, and chronic ischemic nephropathy, described by pathological remodelling of kidney tissue, ultimately leading to kidney injury and chronic kidney disease.

## 1. Introduction

Renovascular disease is a complex disorder of the renal vascular bed. In broad terms, renovascular disease is an entity characterised by the progressive narrowing of the renal arterial or venous vessels, presenting with multiple symptoms ranging from scantly symptomatic disorders and renovascular hypertension to prerenal acute kidney injury (AKI) or ischemic nephropathy, leading to irreversible kidney damage and contributing to chronic kidney injury [1]. In practice, this term is most often equated with stenosis of the renal artery, and conditions solely affecting renal veins are rarer. Renal artery stenosis (RAS) is a primary disease involving the large and medium arteries, whereas secondary vascular diseases develop in intrarenal, small vessels [2]. RAS is conditioned mainly by two disturbances: fibromuscular dysplasia or atherosclerosis of the renal artery. These entities are unilateral (unilateral atherosclerotic renal artery stenosis, unilateral fibromuscular dysplasia, arterial embolus) or bilateral (bilateral atherosclerotic renal artery stenosis), and RAS may also occur as renal artery stenosis to a solitary kidney [1].

Fibromuscular dysplasia (FMD) is a non-inflammatory and non-atherosclerotic malformation of the vasculature [1,2,3]. It is estimated that FMD is in 20–40% of cases responsible for RAS [4]. FMD may affect various arteries (e.g., carotid or vertebral), including renal ones, and leads to arterial stenosis, aneurysm formation with dissection and/or occlusion of medium-size vessels. Anatomically and according to angiographic appearance, FMD occurs in three types: medial (accounting for 85–90% of all FMD cases), intimal and adventitial or periarterial [2,3,4,5]. Although FMD is observed to progress to higher degrees of renal artery stenosis, it rarely causes total occlusion and ischemic atrophy of the affected kidney [4]. The vascular lesions are located away from the origin of the renal artery and are usually found in the midportion of the vessel or at the first arterial bifurcation [1]. FMD almost exclusively affects women between 30–50 years of age, which suggests that hormonal influences may be essential in FMD pathophysiology. However, it is not fully understood, and different aspects, including smoking, family prevalence of some diseases (pheochromocytoma, Ehler’s-Danlos syndrome type IV, Alport’s syndrome, cystic medial necrosis, coarctation of the aorta), a genetic predisposition that weakens the integrity of the vascular wall, or treatment with some drugs (ergotamine preparations, methysergide) have been suggested [2,3,6].

The literature on the genetics of FMD is still scarce, and is lacking unambiguous findings. According to pathophysiological rationale, it could be expected that patients with congenital diseases of connective tissue, manifesting by include arterial stenosis, tortuosity, aneurysms and dissections, are predisposed to FMD development and the consequent potential initiation of RAS. The expected inherited abnormalities predisposing to FMD are based on the presence of variants in genes controlling pathways related to the extracellular matrix of the arterial wall. However, genetic screening of gene disorders in patients with congenital CTD in relation to genes *COL3A1, FBN1, PLOD1, TGFbR1, TGFbR2, TGFb2, SMAD3, ACTA2,* and *COL5A1* was negative. Therefore, while FMD and CTD share some similarities, common genetic backgrounds could not be found [7]. Similarly, the findings of single nucleotide polymorphisms in the elastin gene (*ELN*), as well as in the alpha1-antitrypsin gene (*AAT*) [7,8] and the actin-2 gene (*ACTA2*) [7,9], were disappointing, since they were distributed evenly in patients with FMD but also in healthy controls and in patients with essential hypertension. This could have been due to the modest sample size and lack of comprehensive coverage of the genes of interest, including regulatory regions.

An interesting finding was from Kiando et al. [10], who found that allele A of a genetic variant (rs9349379) of the phosphatase and actin regulator 1 gene (*PHACTR1*), highly prevalent in the general population (~60%), was associated with a 40% increased risk of FMD.

To sum up, taking into account the high prevalence of asymptomatic FMD (~3–6%) and the influence of environmental modifiers (e.g., female hormones, lifetime mechanical stress, tobacco use), a complex genetic basis for FMD is suspected, and the hypothesis provides a rationale for further genetic association studies [11].

Atherosclerotic renal artery stenosis (ARAS) is predominantly seen in older patients as a part of systemic atherosclerosis and the presence of atherosclerotic changes in the abdominal aorta. ARAS is diagnosed mostly in men (male: female is 2:1) older than 50–55 years. The renal atherosclerotic plaques are commonly bilateral and present as eccentric or concentric lesions located approximately 1 cm from the ostium (ostial plaques) or in the proximal one-third of the renal artery. Unlike FMD, atherosclerotic changes in the renal vessels often cause total obstruction of the renal artery and subsequent severe ischemic complications [4]. Patients often present characteristic risk factors: diabetes, hypertension, dyslipidemia, smoking history, peripheral vascular disease, and coronary syndromes [1,4,6].

Moreover, similarly to FD, there is some genetic background predisposing to the development of ARAS. A significantly higher frequency of the angiotensin-converting enzyme (ACE) gene *ACE-D* allele when compared to matched control subjects was demonstrated in ARAS patients. The presence of the *ACE-D* allele was also revealed in other disturbances, including cardiac hypertrophy and remodelling, ischemic or idiopathic dilated cardiomyopathy, hypertrophic cardiomyopathy, and diabetic nephropathy. The mechanism responsible for increased risk of renal artery disease in patients with *ACE-D* allele may be linked to the overexpression of active angiotensin-converting enzyme in plasma or on endothelial cells (contributing to elevation of circulating and/or local angiotensin II levels and to reduction in bradykinin level). Alternatively, RAS development could be due to products expressed by a culprid gene or by several genes in linkage disequilibrium with the ACE gene. Another genetic variant that could have a bearing on the renal vasculature is the angiotensin II type 1 receptor A1166C (AT1R A1166C) polymorphism. Patients homozygous for the C allele of this polymorphism have increased sensitivity to angiotensin II and may, in combination with the ACE-D allelle, have increased risk of cardiovascular complications [12]. There are also reports of a polymorphism in the endothelial nitric oxide (eNOS) gene. The gene encoding eNOS has a G-to-T polymorphism at position 894, leading to the substitution of Glu by Asp at codon 298 of the eNOS protein. This variant is associated with a reduced response to the eNOS inhibitor L-NMMA in healthy volunteers and may be related to the occurrence of atherosclerotic complications [13].

The other RAS causative factors are uncommon and involve extra- and intrarenal disturbances. They are listed in Table 1.

The clinical significance of RAS depends on the location of the stenotic changes and the degree of narrowing of the renal artery. There are many classifications regarding the advancement of the decrease in the renal artery lumen. Stenosis less than 50% is considered to be mild and results in no significant reduction in renal blood flow (RBF); thus, it is not associated with impairment of renal function. Stenosis greater than 50–60% causes a pressure gradient greater than 15–20 mmHg, which is a haemodynamically significant feature of renal stenosis and a possible factor initiating renovascular hypertension development [6]. It results from the pathophysiological premises. The blood flow (Q) in the renal artery, as in other vascular beds, is directly proportional to the perfusion pressure (dP) and inversely proportional to the flow resistance (R). According to what is known in physiology as Poiseuille’s law, R is directly proportional to the length of the stenotic region and inversely proportional to the fourth power of the vascular radius. Thus, the double radius reduction of the patent vessel causes an eight-fold increase in flow resistance, which generates the perfusion pressure increase in this area [14].

**Figure 1:**
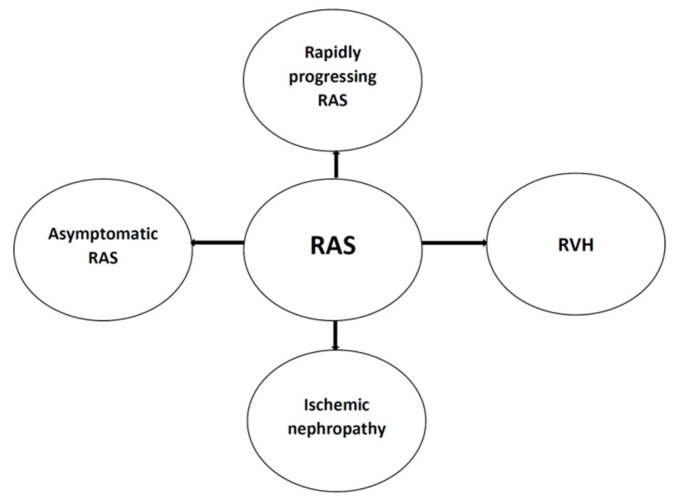
The potential renal artery (RAS) clinical scenario. In some cases, this disorder is asymptomatic due to the fact that the narrowing of the renal artery is haemodynamically insignificant. RAS may be mainly determined by the development of renovascular hypertension (RVH) and its complications. The long-term progression of RAS may also result in ischemic nephropathy development, contributing to subsequent chronic kidney disease. The rapid progression of RAS, reaching critical renal artery stenosis, is a possible aetiological factor of prerenal acute kidney injury (AKI).

The consequences of RAS are manifold, and, as mentioned in the Introduction, RAS may be a solely asymptomatic disorder or it may be accompanied by renovascular hypertension (RVH), eventually developing further consequences. There is also a link between RAS and ischemic nephropathy, even leading to kidney fibrosis and chronic kidney disease (CKD) development. Taking into account the dynamics of RAS development, the rapid progression may be distinguished, with acute nephropathy resulting in acute prerenal form of AKI. On the other hand, the slow progression of RAS and the relative impairment of RBF triggers mechanisms leading to chronic inflammation and fibrosis, contributing to the development of CKD [1,2,3,4]. The possible scenarios are presented in Figure 1.

In sum, renal artery stenosis, characterised by a haemodynamically significant narrowing of the renal artery, produces renovascular hypertension, which may coexist with further complications arising from ischemic nephropathy development. It must be highlighted, therefore, that RAS is not a simple synonym for RVH—RAS is an anatomical diagnosis (clinically, usually taken into consideration when there is a ≥ 75% narrowing of the diameter of a main renal artery or > 50% luminal narrowing with a poststenotic dilatation), and many lesions identified with imaging studies, mostly in elderly patients, remain clinically insignificant [1,15]. The RVH may be diagnosed correctly and properly retrospectively, based on the patient’s response occurring 6–12 weeks after the implemented intervention aimed at the pharmacological or surgical methods alleviating the consequences of RAS, contributing to the elevation of the blood pressure [4,6,15]. There are also some characteristic clinical features suggesting RVH diagnosis, both in anamnesis and examination of the patient. They are listed in Table 2.

The diagnosis and evaluation of RAS is based on the imaging diagnostic modalities. The diagnostic gold standard for the identification of RAS is angiography; however, the technique is invasive and should not be used as an initial diagnostic test [16,17]. Duplex ultra­sonography, which combines direct visualization of renal arteries with Doppler velocity measurements of blood flow, is an excellent screening test for RAS since it is non-toxic and involves no exposure to ionizing radiation [18]. The next diagnostic tools are magnetic resonance angiography (MRA) with gadolinium con­trast media and computerized tomographic angiography (CTA). Both of these imaging techniques are non-invasive and can visualize RAS utilizing computer software to reconstruct an image from X-rays projected from several directions targeted at the same vessel of interest (CTA) or to use the property of nuclear magnetic resonance allowing for the visualization of the target vessel structure without the need for ionizing radiation (MRA) [19]. Although both CTA and MRA are more accurate compared to ultrasonography, they have the potential of kidney damage, and when physicians order contrast CT angiography or MRA, glomerular filtration rate (GFR) must be calculated and patients must be counseled on the risks of nephrotoxicity [16].

As with all forms of hypertension, the overall goal of managing RVH is to reduce the morbidity and mortality associated with elevated BP [20]. In patients with ARAS, invariably associated with systemic atherosclerosis, atherosclerotic risk factor must be controlled, and lifestyle modification must be implemented, including smoking cessation. Pharmacotherapy is focused on target-level driven control of blood pressure, and glycaemic and lipid levels, together with intensive multi-targeted vascular protective therapy [21]. ACE inhibitors (ACEI), angiotensin II AT1 receptor blockers (“sartans”), calcium channel blockers, diuretics (thiazides) and beta-blockers are indicated for the treatment of RAS-related hypertension. Patients with ARAS should also routinely be treated with statin and antiplatelet therapy. ACE inhibitors/angiotensin receptor blockers are a mainstay of RAS treatment. However, the modulation of the renin–angiotensin system in patients with RAS secondary to solitary functioning kidney or severe bilateral stenoses has the potential to dramatically decrease GFR and induce AKI and to reduce perfusion pressure so low as to induce ischemic nephropathy in pressure-dependent kidneys. Therefore, ACE inhibitors and sartans are contraindicated in those patients [20]. Some drawbacks of pharmacotherapy and the presence of patients with severe RAS refractory to the abovementioned hypotensive drugs were the impetus for the development of more permanent and decisive solutions such as surgical and percutaneous revascularization. Surgical restoration of renal blood flow became practical in the 1960s, and it can be accomplished using bypass grafts, aortorenal or nonanatomic, and the more technically challenging aortorenal endarterectomy. There are also endovascular interventional techniques (percutaneous transluminal balloon angioplasty, stenting), which have been a first-line invasive treatment of RAS in the large majority of patients with high-grade ARAS and FMD, with cardiac destabilization symptoms including recurrent or sudden-onset, “flash”, pulmonary edema with severe hypertension [22]. Moreover, patients with ARAS are most likely to benefit from renal artery stent placement since it is associated with greater long-term patency than angioplasty alone [22,23].

The details of aspects of diagnosis and treatment of RVH are out of the scope of the review and may be found elsewhere, including the papers indicated above.

## 2. Pathophysiology of Renovascular Hypertension

### 2.1. The Role of the Renin–Angiotensin–Aldosterone System 

As mentioned above, the most important pathophysiological issue related to RAS is RVH development. As the RAS progress and renal flow decrease steeply, reaching a significant level, it initiates mechanisms responsible for RVH development. In the general RVH pathophysiology, a key role plays the activation of the renin–angiotensin–aldosterone system (RAAS) and retention of sodium and water. 

RAAS is one of the essential mechanisms responsible for both the rapid control of blood pressure (acting synergistically with the sympathetic nervous system) and the circadian or even long-term control of the blood pressure by affecting vascular resistance [24]. The other important determinant of blood pressure includes pressure–volume regulation, according to Guyton’s hypothesis [25] based on the dependence on the pressure and natriuresis. 

RAAS is crucial in blood pressure homeostasis regulation in the vast majority of animals. Considering evolutionary changes, it was shown that the basic components of RAAS appeared in lungfish around 420 million years ago. The essential genes of the RAAS pathway, encoding renin, angiotensinogen, angiotensin-converting enzyme, angiotensin II receptor 1 or aldosterone synthase appeared in the human genome about 0.2 million years ago [24].

The research on RAAS was initiated in the mid-nineteenth century, and the triggering point was the observation that patients with kidney disease and a long duration of coexisting hypertension had an enlarged heart muscle. Therefore, a strong dependence between hypertension and kidney dysfunction was revealed and experimentally confirmed by Robert Tigerstedt and Per Bergman, who evoked a marked rise in blood pressure in rabbits who received injections with kidney extracts. Therefore, they concluded that the kidneys must produce some factor responsible for the observed rise in blood pressure [26]. Another important work contributing to the discovery of the RAAS was the research of John Loesch, who for the first time noticed that prolonged kidney ischemia causes an increase in blood pressure, and, at the same time, rapid reperfusion gradually normalises its value, although his experiment, published in a German-language journal, did not gain as much publicity as the work of Harry Goldblatt, who in similar years also studied the influence of kidney ischemia on the regulation of blood pressure [27]. In his famous experiment, Goldblatt partially constricted the major renal arteries in dogs using adjustable silver clamps. The manoeuvre resulted in reproductive and persistent blood pressure increase, and the clamping of other large arteries (e.g., splenic or femoral) did not produce hypertension. Thus, Goldblatt confirmed Loesch’s previous reports, and these results were premises for the search for a factor of renal origin present in peripheral blood capable of increasing the blood pressure [26,28]. The final isolation of renin is owed to another group of researchers led by Eduardo Braun-Menendez and Irvine Page, who after many attempts discovered a protein with the nature of a plasma enzyme, initially called “hypertensin” or “angiotonin”. Furthermore, Leonard Skeggs et al. contributed to the currently recognised description of RAAS by identifying the substrate for renin, angiotensinogen, and suggesting the existence of a cascade of changes in this relationship [26,27,28]. The contemporary functional description of RAAS is based on the proteolytic role of renin released in response to a decrease in renal blood flow or a decrease in the sodium load flowing to the chemoreceptors of the kidneys (macula densa), which secondarily leads to the formation of angiotensin peptides and aldosterone secretion.

The renin released from the juxtaglomerular apparatus (JG; a group of the baroreceptors named granular cells, located mainly in the walls of the afferent arterioles that deliver blood to the glomerulus) converts the circulating prohormone synthesised by the liver, angiotensinogen, into angiotensin I [1,4,5,6]. Then, the latter peptide is converted by the angiotensin-converting enzyme, which is expressed strongly in many types of endothelial cells, especially in the pulmonary capillaries, as well as in epithelial cells in the renal proximal tubules, small intestine and epididymis, into angiotensin II (AII), a potent vasoconstrictive agent [29]. Angiotensin II action is a causative factor responsible for the increase in the total peripheral resistance, which is the main cause of blood pressure elevation. The developing hypertension is also conditioned by an increased sodium and water reabsorption, resulting from AII activation of the Na^+^/H^+^ antiporter in the proximal tubules and vasoconstriction of the vasa recta in the loop of the Henley. The vasoconstriction and fluid retention is further augmented by aldosterone released in an AII-dependent mechanism from the glomerular layer of the adrenal cortex [6]. The main AII synthesis route described above is also accompanied by “side branches” in which other angiotensin peptides are formed, including the heptapeptide angiotensin 1-7 (antagonising the effects of AII) and central angiotensin III and IV, with the participation of the angiotensin-converting enzyme II and non-specific enzymes like carboxylases, cathepsins and chymases. A detailed description of the structure of the RAAS and the molecular aspects of the functioning of this system is out of the scope of the review and can be found in other papers focusing on these issues [30,31,32]. The above-mentioned AII action is mediated by the AT1 receptors. It was demonstrated in experimental studies that AT1 receptor-knockout mice do not develop hypertension; thus, AT1 receptors exert a crucial role in blood pressure control [1,33]. It must also be highlighted that AII-evoked, preferential vasoconstriction action on the efferent arterioles in the stenotic kidney contributes to the maintenance of GFR despite the reduction of RBF. Thus, the removal of AII after angiotensin-converting enzyme inhibitors or angiotensin II receptor AT1 antagonists (“sartans”) can dramatically decrease GFR in patients treated with those drugs. In the presence of a still-functioning contralateral kidney, GFR disturbances may be scant. At the same time, ACEI/ARB therapy must be carried out with particular caution in patients with bilateral RAS or patients with solitary kidney stenosis due to the possibility of a rapid deterioration of kidney function and the occurrence of oliguria, according to the mechanism mentioned above [1].

**Figure 2:**
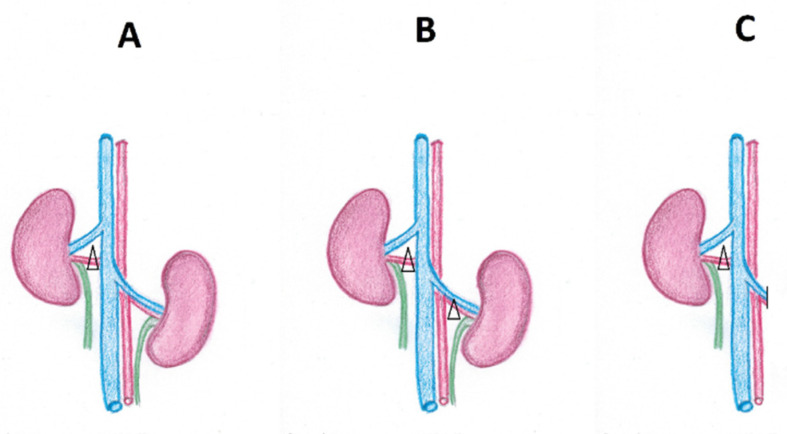
Three potential scenarios of renal artery stenosis: (**A**) model 2K1C: 2 kidneys present, with unilateral stenosis (one kidney is clipped in the experimental studies); (**B**) model 2K2C: 2 kidneys present with bilateral stenosis (both kidneys are clipped in the experimental studies); (**C**) model 1K1C: a solitary, stenotic (clipped in the experimental studies) kidney present. The clip is marked with a triangle.

It should be emphasised that the detailed pathophysiology of RVH depends on the type of RAS. In experimental studies, three models (A-C) were distinguished, corresponding to the clinical encountered scenarios: (A) model 2K1C—two kidneys present, with one affected with stenosis (a clip in the experimental study); (B) model 2K2C—two kidneys present, both affected with stenosis (both kidneys clipped); (C) model 1K1C: a solitary, stenotic (clipped) kidney present. The abovementioned possibilities are illustrated in Figure 2.

In the 2K1C model, reflecting unilateral RAS, the most common scenario observed in clinical practice, the first phase, the initiation phase, is renin-dependent, and the RAAS action predominates in the initiation of blood pressure increase. In the early stage, RAAS action becomes the most important determinant of hypertension development. The pharmacological RAAS blockade or removal of the stenotic kidney promptly normalises the blood pressure. Then, a transition phase occurs, and both kidneys exert opposite effects—the stenotic, ischemic kidney produces renin, which maintains the high hypertensive activity of RAAS, while the contralateral kidney decreases secretion of the renin and increases pressure natriuresis; thus reducing the pressor effects of RAAS. It can be concluded, therefore, that the state of a new dynamic equilibrium is established, namely arterial pressure allostasis. The arterial pressure remains at a higher level, with reduced plasma renin activity (“reverse tachyphylaxis”) due to reduced renin secretion by the compensating kidney. The duration of this phase is individually variable and depends on the compensatory capacity of the contralateral kidney. Administration of angiotensin-converting enzyme inhibitors or angiotensin II receptor antagonists or removal of a stenotic kidney can still normalise hypertension, although this effect is not marked as quickly and clearly as in the initiation phase. Finally, phase III (chronic) occurs, characterised by the failure of the non-stenotic (unclipping) kidney to lower blood pressure. The progressive increase in the hydrostatic intra-glomerular pressure of the contralateral kidney due to the enhanced pressure natriuresis contributes to its dysfunction. In experimental studies using the chronic 2K1C model, the weight and dimensions tend to be lower compared to sham control, suggesting the development of the atrophy of the kidney [4,34]. The role of AII in maintaining hypertension in this phase is not clear, as both the plasma level of the peptide and plasma renin activity may be near normal. Moreover, in the chronic phase, a disproportionately high level of AII was found in the kidney tissue, which results both from the progressive endocytosis of this compound from the circulation during the initiation phase and from the intrarenal synthesis of AII in the contralateral kidney. The intrarenal renin–angiotensin system (mostly located in secretory granules around the glomerular vessels and macula densa) has been demonstrated in several studies already in the 1990s of the last century [35,36,37]. Taking into account the intrarenal AII synthesis, a gradient of AII concentration in kidney and plasma should favour the diffusion of AII from the kidney to plasma in this phase. However, the opposite occurs: there is a renal uptake of AII from plasma with a parallel increase in intrarenal RAAS activity, and AII also brings into action some secondary mechanisms that maintain hypertension via other than general vasoconstriction mechanisms, briefly described below [38]. The increased renal clearance of AII from plasma was revealed by both Navar et al. [39] and Van Kats et al. [40] in their experimental studies based on the assessment of plasma and intrarenal, radiolabelled AII levels. They also showed a higher endogenous AII amount in the non-stenotic kidney than it would appear from the given exogenous, radioisotope labelled AII dose. This suggests an overactivation of intrarenal RAAS, and the increased synthesis of AII in the kidney that is so far compensating hypertension, in contrast to the stenotic one, may also be triggered by the renin-independent mechanism. In some experimental studies, a strong ACE staining was demonstrated in renal sections from a non-clipped kidney of hypertensive rats and dogs, and the increased ACE activity was co-responsible for the elevated level of intrarenal AII in this kidney. The increased ACE presence was correlated with the increased infiltration of kidney interstitial by the inflammatory cells [41]. The abovementioned phenomena eliminate the compensatory function of the kidney without stenosis, and henceforth, both kidneys begin to sustain hypertension. In sum, the early phase of hypertension in the 2K1C model is characterised by AII-dependent, high-vascular resistance with secondary hyperaldosteronism development. In contrast, the chronic phase of this disorder is hypertension resulting from sodium and water retention and expansion of the extracellular space. The remaining two models (2K2C and 1K1C) may be considered as pure sodium- and water-overload-associated hypertension models due to the lack of an initial functional kidney and a compensatory phase [4,34]. The link between sodium (and consequently water) retention and hypertension had already been proven in the 1970s in a famous experiment by Dale, using the salt-sensitive (S) rats and salt-resistant (R) ones. The S rats developed hypertension in response to salt feeding, whereas R animals remained normotensive despite receiving high-sodium diets. Moreover, the transplantation of the S kidney to the R animal resulted in the development of hypertension, while the replacement of the S kidney by the R kidney prevented its development [42]. Since then, a large amount of both experimental and clinical studies confirmed the strong relationship between high salt intake and high blood pressure, as well as the relationship between potassium depletion and the development of hypertension. Some of them are discussed in the review by Rossier et al. [24]. The current research also reveals some detailed, secondary issues related to the age-dependent pathophysiological differences in Dahl’s experimental model. Hypertension developed in young Dahl rats is determined by an evident sympathetic overactivity accompanied by nitric oxide deficiency, abnormal baroreceptor response and profound increase in vascular peripheral resistance. On the contrary, salt-dependent hypertension in adult rats seems to be characterised by augmented oxidative stress and endothelin action. A wide summary of the fifty years of research about the salt contribution in hypertension can be found in a review by Zicha et al. [43].

### 2.2. The Participation of Functional Mechanisms Functionally Associated with RAAS 

The operation of the RAAS and the sympathetic nervous system is closely related. Moreover, RAAS is functionally closely linked with the endothelin system and the kallikrein–kinin system.

Endothelin (ET) was discovered in 1988 as the greatest vasoconstrictive factor of any known endogenous compound and is synthesised by endothelial cells. It is now known that, apart from its vasoconstrictor action, endothelin exerts multiple biological functions via autocrine, paracrine and endocrine signalling pathways, including cell growth and proliferation and extracellular matrix accumulation [44]. ET involves a family of three peptides (ET 1–3), with predominant ET1 form, acting via ETA or ETB receptors. ET is integral to cardiovascular physiology and pathophysiology. The binding of ETA receptor located on the vascular smooth muscle cells leads to elevation of blood pressure due to vasoconstriction, mesangial proliferation, and release of catecholamines, which enhance the pressor effects evoked by ET.

On the contrary, ETB activation causes anti-hypertensive effects via vasodilatation, conditioned by increased prostacyclin and nitric oxide production in vascular endothelium as well as via increased natriuresis and anti-mitogenic effects in vascular walls [45,46]. ET 1 is also present in the kidney, produced by mesangial and glomerular epithelial cells and renal tubular and medullary collecting duct cells. Both ETA and ETB receptors are also expressed in kidneys. In the glomerulus, the ETA receptor was found to be more expressed in podocytes, while the other parts of the glomerulus show a similar presence of both receptors. In renal vasculature, vascular smooth muscles of afferent and efferent arterioles expressed mostly ETA receptors, whereas ETB ones were demonstrated on endothelial cells of afferent and efferent arterioles. Proximal tubules and Henle’s loop express both ETA and ETB receptors; however, their density seems to be low. The highest ET receptors expression was confirmed in collective ducts, with ETB receptor as the predominant one. ET acting via ETA kidney receptors promotes vasoconstriction, podocyte damage and fibrosis, while activation of ETB receptors results in vasodilator, antiproliferative and antifibrotic effects [45,46]. The detailed characteristics of the endothelin system and its importance in human physiology and pathophysiology can be found in numerous studies on this issue [47,48,49,50,51], along with a discussion focusing on the pharmacological benefits of using endothelin receptor antagonists in currently applied and future potential pharmacological management of important cardiovascular (essential hypertension, pulmonary arterial hypertension, cancer, chronic pain) and renal (chronic kidney disease, diabetic nephropathy) diseases [52,53].

Both the endothelin system and RAAS are functionally dependent. An angiotensin II-induced gene transcription upregulates ET synthesis. In turn, ET-1 increases the secretion of other vasoconstrictors, including AII [45,54,55]. In an experimental study, rats infused with AII presented overactivity of ETA receptors, and the action of ETB ones was reduced [56]. Thereby, a positive feedback loop between RAAS and endothelin system exists, and an inherent and inevitable consequence of RAAS activation occurring in the course of RAS must be the co-activation of the endothelin system.

A similar functional relationship exists for the RAAS and the other system exerting its action through autocrine, paracrine, and neuroendocrine-mediated effects, namely the kallikrein–kinin system (KKS). The KKS is a cascade of plasma and tissue enzymes (serine proteases; e.g., kallikrein, plasmin) and precursor kininogen proteins (Fitzgerald factor—high-molecular-weight kininogen, low-molecular-weight kininogen and the Hageman factor, factor XII), the activation of which leads to intravascular activation of the intrinsic coagulation pathway and enzymatic breakdown of endogenous kininogens, with the consequent release of bradykinin (BK) and other BK-related peptides (e.g., kallidin) [57,58]. KKS was discovered in 1909 as a result of the observation that the component present in the urine (identified in 1930 as HWMK) is able to lower the blood pressure [59]. BK, the main acting compound of KKS, is a potent vasodilatator and a key pro-inflammatory factor, accounting for sustained inflammatory response and edemas formation. It is also involved in activation of the complement system, pain inducement, coagulation and fibrinolysis, cell growth and differentiation, and angiogenesis. KKS acts via at least two kinin receptors: B1 and B2. The latter one is constitutive and mediates most of the physiological effects exerted by BK, and the expression of the B1 receptor increases during inflammation and tissue damage, endotoxin or anoxia. Kinins, as effectors of KKS, are rapidly enzymatically broken down into inactive products with the participation of commonly found tissues and plasma kininases, including aminopeptidases, carboxypeptidases and neutral endopeptidase, but also angiotensin-converting enzyme, which has bifunctional activity in both RAAS and KKS.

Taking into account the premises relating to the physiological and pathophysiological role of KKS, the numerous possibilities of pharmacological intervention based on the interference in the KKS (e.g., in the treatment of inflammation, sepsis, allergic reactions, hereditary angioedema, pulmonary embolism or acute respiratory distress syndrome) are being investigated [57,58,59]. Further detailed information on the organisation, physiology and pathophysiology, as well as the potential therapeutic approach of the KKS system, can be found in numerous reviews [60,61,62,63]. Due to the significant vasodilatory effect produced by BK, conditioned by its own action and potentiated by the secondary release of other vasoactive autacoids, such as eicosanoids, nitric oxide, endothelium-derived hyperpolarising factor and tissue plasminogen activator, KKS play an important role in the regulation of cardiovascular and renal functions. The entire KKS is also present in the kidneys, and intrarenal BK synthetisation is an important component of blood flow regulation, particularly in the kidney medulla. In an experimental study, it was demonstrated that the administration of kinin antagonist into the renal artery decreased RBF and impaired the regulation of the GFR [58]. Considering the fact that RAAS and KKS are functionally related (ACE is a kininase; kallikrein contributes to the increased synthesis of renin initiating the production of AII) [59,64], RAAS activation occurring in response to haemodynamically significant RAS consequently also affects KKS functioning.

### 2.3. The Sympathetic Nervous System

The sympathetic nervous system (SNS) exerts powerful pressor effects, and it is one of the key elements contributing to the complex pathogenesis of essential hypertension. There is also evidence for sympathetic activation in RVH, resulting from disturbed afferent signals from the underperfused kidney. Clinical and laboratory studies, as well as experimental ones, confirmed the sympathetic overactivity in patients and animals with established RVH, respectively. In rats with both 2K1C and 1K1C models, an elevated plasma noradrenaline (NA) level was found. In addition, in 2K2C animals, plasma catecholamine level and noradrenaline spillover were increased.

Similarly, increased plasma NA concentration and increased urinary excretion of catecholamine metabolites were reported in RVH patients. Plasma NA assessment is regarded to be an indirect biochemical marker of sympathetic activity, and its spillover into the peripheral blood depends on many factors, e.g., the rate of release from neuronal terminals, synaptic clearance, specific or unspecific reuptake by nerve endings or non-neuronal cells, respectively, and enzymatic degradation in blood. Despite all of the reservations related to the interpretation of the NA load in circulation and the absence of any direct and precise method of SNS activity quantification, plasma NA measurement along with urinary catecholamine metabolites evaluation are still non-invasive means of assessing the sympathetic activity [65,66]. There are also clinical reports demonstrating increased muscle sympathetic nerve activity (MSNA), and the results of experimental studies confirm the increase of direct renal sympathetic nerve activity (RSNA) in RVH [66]. The next important finding is that in RVH patients, the decreased baroreflex sensitivity was shown. Experimental studies demonstrated that AII impaired baroreflex bradycardia and heart rate response to injection of phenylephrine and nitroglycerine. RVH patients were characterised by similar baroreflex dysfunction compared to those observed in severe essential hypertensive ones, and baroreceptor heart rate control in both primary hypertension and RVH was displaced toward elevated blood pressure values [67].

It should be emphasised that AII is the key mediator contributing to the overactivation of SNS stemming from both AII-related activation of the postganglionic sympathetic neurons with modulation of the baroreflex properties and AII action on the central nervous system. Campos et al. [68] in their experimental study demonstrated that the sympathoexcitation in RVH animals resulted from increased activity of paraventricular nucleus of the hypothalamus and the rostral ventrolateral medulla, the regions of the nervous system mostly involved in the central control of the sympathetic vasomotor tone. In sum, the pathogenesis of enhanced sympathetic activity in the course of RAS is associated with RVH development. It is conditioned by a dysfunction in the cardiovascular reflexes (resulting from abnormal arterial baroreceptors, the volume receptors and chemoreceptors), which in physiological conditions are the main restrainers of tonic sympathetic drive, and an adrenergic augmentation induced by various agents (angiotensin II, endothelin), which exert excitatory effects on sympathetic neural function at both peripheral and central level [69].

### 2.4. The Recruitment of Additional Mechanisms

The effects of AII, the key effector of RAAS, which is overactivated in RAS, clearly extend beyond the most important ones—vasoconstriction and sodium retention and consequent blood pressure elevation. The compound is also considered to induce and sustain inflammatory and fibrogenic mechanisms. AII exerts a potent pro-inflammatory effect, and it has been indirectly shown that the positive effect of ACEI and ARB, commonly used in cardiovascular diseases (hypertension, chronic heart failure), is partly due to their anti-inflammatory properties. Moreover, taking into account the fact that RAS occurs as a part of the atherosclerotic process, the role of AII as a pro-inflammatory mediator promoting the development of atherosclerosis due to the induction of smooth muscle cells proliferation, endothelial injury and dysfunction, increased systemic oxidative stress, a tendency for plaque rupture and inhibition of fibrinolysis, becomes particularly important [1,14]. AII induces the synthesis of pro-inflammatory mediators and the recruitment of inflammatory cells to the regions of damaged vascular endothelium. Moreover, it upregulates the expression of selectins, integrins and other adhesion proteins in endothelial cells and leukocytes and stimulates the intracellular synthesis and release of chemokines such as monocyte chemoattractant protein-1 (MCP1). AII contributes to the indirect stimulation of the inflammatory process and ARAS development through the upregulation of NF-κB in monocytes, macrophages, endothelial cells and vascular smooth muscle, followed by excessive release of TNF-alpha and Il-6, enhancing the immune-inflammatory response [31,70,71]. AII is also a major profibrogenic cytokine attenuating renal interstitial fibrosis through modulation of renal cell growth, synthesis of extracellular matrix and multiple fibrotic pathways. Kidney fibrosis is the final pathological condition resulting from inflammatory, haemodynamic or metabolic renal injury. The stimulatory effect of AII on fibrogenesis is based on the stimulation of the key mediator of kidney fibrosis, namely transforming growth factor-β (TGFβ) and its co-acting molecules: bone morphogenetic protein-7 (BMP7, which has a protective role against TGFβ-mediated fibrosis) and connective tissue growth factor (CTGF, the main potentiating agent of TGFβ-induced fibrosis). The other profibrotic factors, whose synthesis is increased by AII, include ET-1, matrix metalloproteinase-2 (MMP-2), tissue inhibitor of metalloproteinase-2 and plasminogen activator inhibitor-1. The important factor contributing to kidney fibrosis in the course of RAS is also hypoxia, since AII also induces the production of hypoxia-inducible factor-1α (HIF-1α), which is the next powerful causative compound of pathological tissue rebuilding and fibrosis [72,73]. It can be concluded, therefore, that various injury, including impairment of RBF triggering RAAS overactivity in the course of RAS, results in an inflammatory cascade with activation of inflammatory cells and damage of the renal epithelial cells. These cells are the source of inflammatory but also profibrotic mediators that result in consecutive epithelial dedifferentiation. The renal mesenchymal cells (fibrocytes, fibroblasts, pericytes) and tubular and endothelial undergo trans-differentiation, becoming contractile myofibroblasts that start to produce an excessive extracellular matrix [74].

The disturbed activity of the next important pro-inflammatory and vasoactive mediators acting via autocrine and paracrine manner, i.e., the prostaglandin system, should also be mentioned in the context of complex RAS pathophysiology. The participation of the prostaglandins in RAS pathophysiology indirectly results from the fact that some action determined by bradykinin is mediated by the prostacyclin [75]. The prostaglandin system is, therefore, the next functional network, closely related to RAAS and kinins, and the changes in RAAS activity need to be considered in the broad context of the potential impact on other components. There is evidence that cyclooxygenase-2 (COX-2) is expressed in the kidney, mainly in the macula densa (which are differentiated tubular epithelial cells, compromising the juxtaglomerular apparatus) and medullary interstitial cells [76,77]. As mentioned above in the RAAS short description, macula densa is involved in renin release in response to the sensing of luminal sodium chloride in the glomerular filtrate, which is dependent on net apical transport, affecting the Na^+^/K^+^/2Cl^−^ cotransporter. The cotransporter has a high affinity for Na^+^ and K^+^ concentrations, and angiotensin II directly stimulates Na^+^/K^+^/2Cl^−^ cotransporter via AT1 apical receptors [76,78]. The conditions with hyperreninemia due to the reduction in sodium chloride amount in ultrafiltrate (e.g., salt deficiency, administration od diuretics or ACEI/ARB) are associated with an increase in the expression and enzymatic activity of COX-2 in the kidneys. On the contrary, AII decreases COX-2 expression within the JG by stimulating the Na^+^/K^+^/2Cl^−^ cotransporter and increasing intracellular sodium chloride concentration [75,79]. In in vitro studies, the use of PGE2 and PGI2 stimulate renin release in cultures of the JG apparatus cells. Moreover, in animal models of RAS, resulting in RBF reduction, renin release and development of RVH, the expression of COX-2 was demonstrated to be upregulated [79]. The role of the particular prostaglandins in RVH development has been studied in the 2K1C model in mice. It was demonstrated that after one week of the 2K1C procedure, the blood pressure was elevated in a wild-type mouse.

In contrast, the animals lacking the prostacyclin IP receptor (IP-/- mice) were characterised by significantly lower values of blood pressure, and expression of renal renin mRNA was significantly lower in IP-/- animals [80]. PGE2 analogue or selective EP agonists administration affected renin mRNA content in cultured JG cells [79]. Other studies demonstrated that PGE2 stimulates renin release via activation of EP2 and EP4receptors, whereas EP1and EP3 receptors appear to be without functional relevance in juxtaglomerular cells [81]. The current explanation for these discrepancies is unknown, and further studies on the role of individual prostanoids in the pathophysiology of RVH in relation to RAS are required. The detailed description of the interactions between COX and RAAS in the kidney is complex, and the discussion of the issue with a summary of the role of the prostaglandins in kidney diseases may be found in some narrative reviews [82,83,84]. In general, COX-2-derivative prostaglandins are believed to counter-regulate the pressor effects of RAAS and to counterbalance the vasoconstriction produced by Ang II. In the kidney, physiologically, both prostanoid systems and RAAS are essential for vasoactive regulation and maintaining a balance between vasodilation and vasoconstriction. However, numerous studies also suggest a pivotal role of their different interplay in pathology. COX-2 increases the renin release and Ang II formation, leading to an increase in blood pressure. On the other hand, COX-2 restricts the Ang II-mediated increases in renovascular resistance by eliciting direct vasodilation produced by PGE2. The findings indicate the potential conversion of beneficial effects of COX-2 produced derivatives, which become a dysfunctional regulator of the RAAS activity and potentiate Ang II-mediated RVH [82].

Increased oxidative stress also accompanies inflammatory and fibrotic disturbances. The phenomenon of oxidative stress is a process of increased formation of reactive oxygen or nitrogen species. Under physiological conditions, there is a continuous synthesis of a small amount of free radicals (free reactive species) during cellular aerobic respiration or local inflammatory responses (with the participation of macrophages). These molecules are formed in the mitochondrial respiratory chain as byproducts during electron passage by a complex of cytochromes (NADPH oxidase, flavoprotein oxidases). Free radicals are regarded to be signalling molecules that play a role in cell differentiation and apoptosis; thus, in the case of acceleration of apoptosis, free radicals contribute to cellular ageing. The physiological generation of free radicals is precisely controlled by enzymatic and non-enzymatic (e.g., antioxidants) defence mechanisms. The increased formation of free radicals is an expression of a dynamic disbalance in the formation and elimination of these molecules in favour of oxidation and diminished antioxidant capacity. The excessive production of free radicals causes damage to the structure of the cells and exacerbates apoptosis and necrosis, thus changing the proper functioning of many cells. Currently, there is no doubt that oxidative stress is an important pathogenetic element in the development of many diseases (including atherosclerosis, neurodegenerative disorders, diabetes, obesity, chronic kidney disease, cancer) and ageing processes and inflammatory conditions [85,86,87]. The oxidative stress is also observed in RAS, and it contributes to the development of RVH in the course of RAS. The phenomenon was confirmed in experimental models of 2K1C and 1K1C animals [88].

Angiotensin II, which is the “central player” in the course of RAS development, stimulates reactive species formation due to the overactivation of NADPH and impairment of mitochondrial function resulting from an accumulation of peroxinitrite, hydroxyl radical and hydrogen peroxide [32,89,90]. It was demonstrated that slow infusion of AII at a rate that does not cause an immediate, strong vasomotor reaction also causes a slowly progressive increase in blood pressure due to the intensification of oxidative stress by this mediator within central nervous system regions that control blood pressure. The studies demonstrated the increased oxidative stress resulted from NADPH overactivity within the paraventricular nucleus of the hypothalamus (PVN) and the rostral ventrolateral medulla (RVLM) of the brainstem, and it was correlated with the central pressor mechanisms. Therefore, it can be concluded that AII-induced increased oxidative stress contributes to an increased sympathetic drive. The possible mechanism by which circulating AII exerts its central effects is by binding to AT1 receptors on neurons in the region of the brain that lack the tight blood–brain barrier (circumventricular organ; CVO). The activation of CVO neurons may transmit the signal to downstream regions of the brain responsible for cardiovascular control (RVLM, PVN). The overactivation of NADPH expressed in the RVLM and PVN and increased free radical formation causes the subsequent increase in the activity of sympathetic premotor neurons [88]. The other mechanisms initiated by the excessive AII-evoked oxidative stress and responsible for hypertension development and maintenance in RVH include reduction of the nitric oxide bioavailability, alternations in the metabolism of arachidonic acid with overproduction of vasoconstrictors and upregulation of endothelin [38]. Oxidative stress was also revealed in the stenotic, clipped kidney, and the chronic, antioxidant treatment downregulated AT1 kidney receptors expression, suggesting that oxidative stress, functionally linked to AII activity, is and important pathophysiological finding in the ischemic kidney [88]. It must also be emphasised that oxidative stress is an essential factor in maintaining high blood pressure in RVH. The dominant role of the RAA system and AII hyperactivity is transient and is the starting point for the initiation of other mechanisms, including excessive oxidative stress. It has been proven in experimental studies of RVH that after a few weeks, hypertension becomes independent of AII, and its course and advancement correlate much better with the markers of oxidative stress [88,91]. The treatment with antioxidants (e.g., superoxide dismutase analogues such as tempol) successfully diminished elevated blood pressure in RVH rats [92,93]. Moreover, studies in humans confirmed the presence of oxidative stress in RVH, and it returned to normal after effective renal artery revascularisation [94]. In sum, AII-induced increased oxidative stress, occurring both in the region of brains involved in blood pressure control and in the stenotic kidney, plays a major role in the maintaining of elevation in blood pressure and overactivity of the sympathetic drive in the advanced RVH phase.

## 3. Ischemic Nephropathy

All the mechanisms mentioned above, present in the course of RAS, lead to the development of RVH but also contribute to kidney damage and stenotic dysfunction. This is called ischemic nephropathy, and this entity may lead to CKD development.

However, the term “ischemic nephropathy” is somewhat misleading since it is not related to a disorder that develops as a result of total occlusion and true kidney ischemia. Kidneys are a relatively overperfused organ when referring to RBF’s metabolic requirements, and high RBF ensures the most important function of the kidneys as a filtering organ. Studies have demonstrated that only about 10% of the oxygen contained in the blood of a perfusing kidney is necessary to maintain the energy demand of this organ [95,96]. Therefore, in a kidney affected by RAS, the blood flow may noticeably fall but without the inevitable threat to the viability of the kidney tissue. Moderate RBF reduction may be sufficient to disturb GFR, and it may achieve a value below the level needed for renal autoregulation of RBF, which results in invariable activation of pressor mechanisms (RAAS activity) and initiation of RVH, with a subsequent diminishing of the kidney size, while maintaining oxygen supply to both the cortex and the medulla of the stenotic kidney and without obvious rebuilding of the kidney tissues [95,97]. Therefore, the term “azotemic renovascular disease” is proposed by some authors as more adequate compared to the commonly used “ischemic nephropathy” for the description of such an entity [98]. The more severe and prolonged diminishing of RBF in the stenotic kidney may threaten the oxygen supply to the organ and viability of the tissues, eventually leading to kidney fibrosis, resulting from action of the key compound, AII, which also exerts strong profibrotic effects, augmented by the secondary release of TGF-β. Since the numerous pathways characterised by TGF-β are part of the complex pathophysiology of atherosclerosis, kidney fibrosis and ischemic nephropathy mostly develop in ARAS patients, and it is rarely observed in those with fibromuscular fibrosis [95]. The process of remodelling of kidney tissues and fibrosis is also characterised by the release of many other inflammatory mediators (e.g., TNF-α, MCP-1) and markers of damage (e.g., neutrophil gelatinase-associated lipocalin-1, NGAL, tissue inhibitor of metalloproteinases-2 (TIMP-2)), and lymphocyte infiltration and macrophages appear in the kidney tissue [99,100]. The inflammatory and profibrotic mechanisms prevailing in advanced RAS contribute to the advanced, pathological remodelling of the kidney structure, which evidence ongoing chronic kidney injury and ultimately account for up to 15–20% of the cases of chronic kidney disease developing [101,102].

## 4. Conclusions

**Figure 3:**
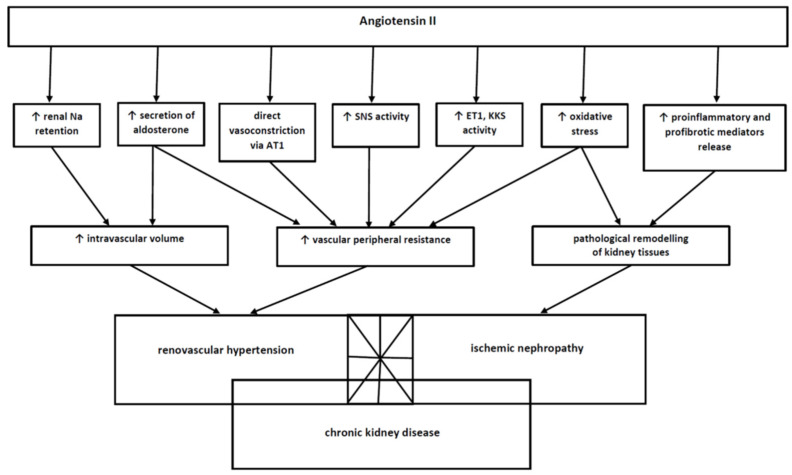
The complex action of angiotensin II and angiotensin II-induced disturbances. The final clinical consequences (renovascular hypertension, ischemic nephropathy) leading to chronic kidney disease are shown in rectangular boxes. The aforementioned disorders may exist in an isolated form. The overlapping rectangular fields indicate the possible coexistence of these diseases.

To sum up, the pathophysiology of renal artery stenosis is complex and includes many pathophysiological mechanisms that are interrelated and dependent on each other. RAS takes the form of an asymptomatic radiological abnormality or manifests with renovascular hypertension and ischemic nephropathy contributing to chronic kidney disease, and these conditions may coexist and overlap, thus widening the potential RAS symptomatology. There is no doubt that the “key player” in the pathogenesis of disturbances developing in the course of renal artery stenosis is angiotensin II, which is a common, connecting pathogenetic element of systems functionally linked with RAAS. They are summarised in Figure 3.

## Figures and Tables

**Table 1:** The causative factors of renal artery stenosis.

	Unilateral Stenosis	Bilateral Stenosis
Lesion of renal vessels (renal artery or its branches or renal veins)	Unilateral atherosclerotic renal artery stenosis Unilateral fibromuscular dysplasia of renal artery (medial, perimedial, intimal) Renal artery aneurysm Renal arterial embolus Posttraumatic segmental arterial occlusion	Bilateral atherosclerotic renal artery stenosis Stenosis to a solitary functioning kidney
Renal parenchymal diseases	Intrarenal compression (carcinoma, sarcoma, metastasis) Extrarenal compression (aortic aneurysm, retroperitoneal hematoma, peripelvic cyst)	
Other clinical entities	Arteriovenous malformations Congenital narrowing Systemic vasculitis Arterial nephrosclerosis Rare diseases (type 1 neurofibromatosis, tuberous sclerosis, Ehler’s-Danlos syndrome, Marfan syndrome)	

**Table 2:** The symptoms suggesting the diagnosis of renovascular hypertension.

Hypertension characteristics	Onset of hypertension in young patients (<30 years); suggestive of FMD Onset of hypertension at and after about 50 years of age; suggestive of ARAS Abrupt onset of hypertension Acceleration of previously well-controlled hypertension Hypertension refractory to an appropriate 3-drug regimen Features of malignant hypertension No family history of hypertension
Renal abnormalities	Unexplained and unprovoked acute kidney injury with/or hypokalemia Acute kidney injury induced by treatment with angiotensin converting enzyme inhibitors or angiotensin II AT1 receptor blockers Unilateral small kidney (asymmetric kidneys with more than 1.5 cm of difference in the size)
Other findings	Continuous, high-pitched holosystolic with diastolic component abdominal bruit or flank Recurrent, unexplained flash pulmonary edema even in the absence of severe congestive heart failure Severe retinopathy (Keith-Wagener-Barker grade III or IV optic fundi) Carotid, coronary vascular disease (suggestive of ARAS) History of cigarette smoking

## Data Availability

Not applicable.

## Abbreviations

ACE	angiotensin converting enzyme
ACEI	angiotensin converting enzyme inhibitors
AII	angiotensin II
AKI	acute kidney injury
ARAS	atherosclerotic renal artery stenosis
ARB	angiotensin II AT1 receptor blockers
BK	bradykinin
CKD	chronic kidney disease
COX-2	cyclooxygenase-2
ET	endothelin
ETA/ETB	endothelin A or B receptors
FMD	fibromuscular dysplasia
GFR	glomerular filtration rate
JG	juxtaglomerular apparatus
KKS	kallikrein-kinin system
NA	noradrenaline
PVN	paraventricular nucleus of the hypothalamus
RAAS	renin–angiotensin–aldosterone system
RAS	renal artery stenosis
RBF	renal blood flow
RVH	renovascular hypertension
RVLM	rostral ventrolateral medulla
SNS	sympathetic nervous system
